# Low-temperature effects on docosahexaenoic acid biosynthesis in *Schizochytrium* sp. TIO01 and its proposed underlying mechanism

**DOI:** 10.1186/s13068-020-01811-y

**Published:** 2020-10-16

**Authors:** Fan Hu, April L. Clevenger, Peng Zheng, Qiongye Huang, Zhaokai Wang

**Affiliations:** 1 Third Institute of Oceanography, Ministry of Natural Resources, Xiamen, 361005 China; 2 Department of Chemistry and Biochemistry, University of Oklahoma, Norman, OK USA; 3 College of Life Science and Health, Wuhan University of Science and Technology, Wuhan, 430065 China

**Keywords:** Low temperature, Polyunsaturated fatty acid, *Schizochytrium*, De novo genome assembly, Differential expressed genes

## Abstract

**Background:**

*Schizochytrium* species are known for their abundant production of docosahexaenoic acid (DHA). Low temperatures can promote the biosynthesis of polyunsaturated fatty acids (PUFAs) in many species. This study investigates low-temperature effects on DHA biosynthesis in *Schizochytrium* sp. TIO01 and its underlying mechanism.

**Results:**

The *Schizochytrium* fatty acid biosynthesis pathway was evaluated based on de novo genome assembly (contig N50 = 2.86 Mb) and iTRAQ-based protein identification. Our findings revealed that desaturases, involved in DHA synthesis via the fatty acid synthase (FAS) pathway, were completely absent. The polyketide synthase (PKS) pathway and the FAS pathway are, respectively, responsible for DHA and saturated fatty acid synthesis in *Schizochytrium*. Analysis of fatty acid composition profiles indicates that low temperature has a significant impact on the production of DHA in *Schizochytrium*, increasing the DHA content from 43 to 65% of total fatty acids. However, the expression levels of PKS pathway genes were not significantly regulated as the DHA content increased. Further, gene expression analysis showed that pathways related to the production of substrates (acetyl-CoA and NADPH) for fatty acid synthesis (the branched-chain amino acid degradation pathway and the pentose phosphate pathway) and genes related to saturated fatty acid biosynthesis (the FAS pathway genes and malic enzyme) were, respectively, upregulated and downregulated. These results indicate that low temperatures increase the DHA content by likely promoting the entry of relatively large amounts of substrates into the PKS pathway.

**Conclusions:**

In this study, we provide genomic, proteomic, and transcriptomic evidence for the fatty acid synthesis pathway in *Schizochytrium* and propose a mechanism by which low temperatures promote the accumulation of DHA in *Schizochytrium*. The high-quality and nearly complete genome sequence of *Schizochytrium* provides a valuable reference for investigating the regulation of polyunsaturated fatty acid biosynthesis and the evolutionary characteristics of *Thraustochytriidae* species.

## Background

Docosahexaenoic acid (DHA, C22:6), a major ω-3 polyunsaturated fatty acid (PUFA), is widely distributed among phospholipids in the human brain and retina, playing a vital role in human health [1]. A variety of marine microorganisms are rich in DHA [2, 3]. As one of the most promising DHA-producing species, *Schizochytrium* has the ability to accumulate DHA at more than 40% of the total fatty acid (TFA) concentration and ~ 40% of the dry cell weight [4]. On account of this characteristic, the PUFA biosynthesis pathway in *Schizochytrium* has attracted increasing attention and is currently being exploited by several companies [5].

The entire PUFA synthesis pathway conventionally occurs via two routes. The first route includes the standard fatty acid synthase (FAS) pathway, which involves serial desaturation and elongation of short saturated fatty acids (SSFAs, C16:0 or C18:0). The second route includes the polyketide synthase (PKS) pathway [6]. In 2002, Metz et al. reported that the PKS pathway is responsible for PUFA synthesis and does not rely on elongation and desaturation of the FAS pathway in *Schizochytrium* through the use of labeling experiments [7]. This result was subsequently validated by Lippmeier et al. who showed that knock-outs of the PKS gene led to PUFA auxotrophic behavior [8]. Although these biochemical and genetic studies on *Schizochytrium* have suggested that the PKS pathway is responsible for DHA biosynthesis [7–9], recent studies revealed that enhancement of the FAS pathway contributes significantly to increased DHA productivity in *Schizochytrium* [10, 11]. Based on these results, questions arise as to which route is employed by *Schizochytrium* for DHA synthesis. Genome sequencing is an ideal approach for investigating this issue, however, information on high-quality whole genomes of *Schizochytrium* currently available in public databases is limited [12], preventing identification of the *Schizochytrium* PUFA biosynthesis pathway.

Low temperatures have been shown to impact PUFA productivity in many PUFA-producing organisms [13–17]. However, few studies have investigated the mechanism underlying these effects. Ma et al. pioneered in-depth studies in *Aurantiochytrium*, showing that the upregulation of the PKS pathway and downregulation of the FAS pathway play important roles in promoting DHA accumulation [13, 18]. Min et al. reported that significant upregulation of the fatty acid desaturases associated with the FAS pathway leads to PUFA accumulation in *Bangia fuscopurpurea* [19]. In *Shewanella piezotlerans* and *Photobacterium profundum*, upregulation of PKS pathway genes was not detected despite the increase in PUFA accumulation at a low temperature [20, 21]. In *Schizochytrium*, Zeng et al. reported that low temperatures have a significant impact on DHA production, increasing DHA levels up to 50% of the TFA level [22]. The mechanism underlying this low-temperature-induced DHA accumulation remains unknown in *Schizochytrium*.

Previously, we isolated a *Schizochytrium* strain from sea water (designated *Schizochytrium* sp. TIO01) [23]. In this study, the effects of low temperature on fatty acid synthesis in *Schizochytrium* sp TIO01 were analyzed and the underlying mechanism investigated. Here, we propose a reconstructed fatty acid biosynthesis pathway of *Schizochytrium* sp. TIO01 based on de novo genome assembly and iTRAQ-based LC-MS-MS. The effects of low temperature on fatty acid synthesis were investigated by differential gene expression analysis.

## Results

### Low temperatures promote DHA accumulation in *Schizochytrium*

**Fig. 1:**
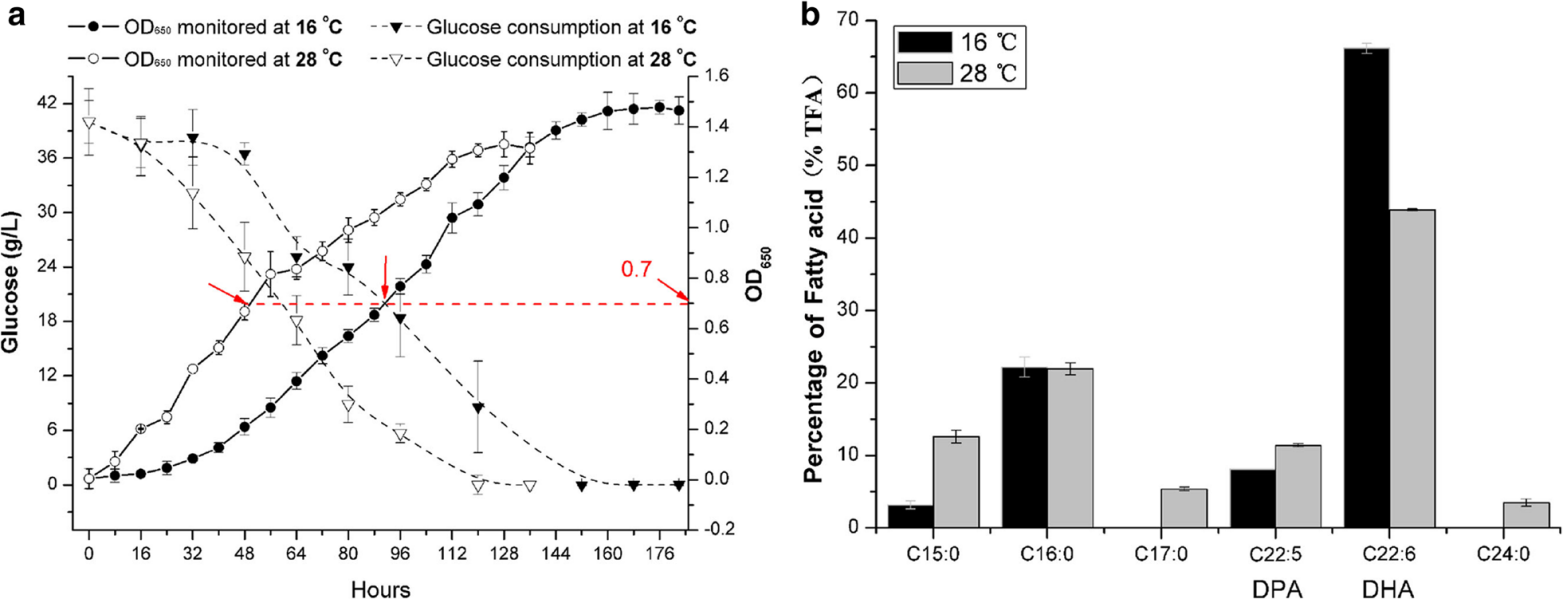
Growth curve (**a**), glucose consumption (**a**), and total fatty acid analysis (**b**) when *Schizochytrium* was cultured under two different temperatures. **a** Time course analysis of *Schizochytrium* growth and glucose consumption. The arrows indicate samples collected for subsequent total fatty acid (TFA) analysis and gene expression analysis. **b** Analysis of the TFA composition of *Schizochytrium*. *DHA* docosahexaenoic acid, *DPA* docosapentaenoic acid

The fatty acid composition profiles of *Schizochytrium* cultured under both cold and normal temperatures were analyzed. Samples used to analyze fatty acids were cultured and collected at an optical density at 650 nm of approximately 0.7 at 28 or 16 °C, as indicated in Fig. 1a. Generally, *Schizochytrium* sp. TIO01 had similar total lipid levels (16 °C: 53.53% ± 2.18% (weight/cell dry weight); 28 °C: 51.05% ± 3.05% (weight/cell dry weight)) and lipid content profiles at the two temperatures. Pentadecanoic acid (C15:0), palmitic acid (C16:0), heptadecanoic acid (C17:0), docosapentaenoic acid (DPA, C22:5, ω-6), and DHA (C22:6, ω-3) were all detected under both conditions. Tetracosanoic acid (C24:0) was detected only when *Schizochytrium* was cultured at 28 °C. At low temperatures, *Schizochytrium* exhibited reduced saturated fatty acid synthesis and a preference for increased DHA synthesis. DHA constituted ~ 65% of the total fatty acid content at 16 °C compared to ~ 43% at 28 °C (Fig. 1b).

### *Schizochytrium* genome assembly, assessment and annotation

To reveal the fatty acid biosynthesis pathways and further improve our understanding of the underlying mechanism by which low temperatures promote DHA accumulation in *Schizochytrium*, we performed de novo whole-genome assembly and annotation. Using 13.9 Gb of PacBio RS II subreads (217 × genomic data) and 31 Gb of Illumina PE250 clean data (480 × genomic data), a genome size of 64 Mb with a 45% GC base ratio containing 34 scaffolds and 3 circular contigs (one of the circular contigs is the mtDNA genome, which was previously reported [24]) was obtained. The N50 scaffold and N50 contig were 5.83 Mb and 2.86 Mb, respectively. Table 1 shows detailed information regarding the assembled *Schizochytrium* genome and the genome for *Thraustochytriaceae* species. To inspect the accuracy of de novo assembly, multiple independent sources of reads were used to assess the assembled genome. Among the 62,457,386 paired Illumina PE250 reads (approximately 480 × coverage of the genome), over 99% could be aligned to the genome. The overall alignment rate of the transcriptomic reads from 14 independent culture conditions (approximately 900 × coverage of the genome) ranged from 95 to 97%, as shown in Additional file 1: Table S1). These results suggest that the assembled *Schizochytrium* genome was of high-quality and nearly complete. We also evaluated the completeness of the genome assembly using BUSCO-v3.0 (database: eukaryota_odb9) [25]. The results showed that 90.4% of the core genes were detected (C: 90.4% [S: 89.1%, D: 1.3%], F: 2.3%, M: 7.3%, 274/303). Based on the integration of RNA-seq data, protein alignment, and de novo predictions, 12,392 protein-coding genes were predicted with an average transcript size of 1876.3 bp and a mean of 1.7 exons per gene. In all, 6796 out of the 12,392 predicted proteins (54.84%) could be classified into families according to their putative functions. The proportions for each database were as follows: KEGG (4362, 35.20%), SwissProt (5078, 40.98%), TrEMBL (6336, 51.13%), Nr (6063, 48.93%), GO (1235, 9.97%), and InterPro (6242, 50.37%).

**Table 1:** Genome comparison among *Thraustochytriaceae* species

General features	*Schizochytrium* TIO01 (This study) (GCA_004764695.1)	*Schizochytrium* M209059 [12] (GCA_000818945.1)	*Aurantiochytrium acetophilum* HS399 [26] (GCA_004332575.1)	*Aurantiochytrium* sp*.* KH105 [27] (GCA_003116975.1)	*Aurantiochytrium* sp. T66 [28] (GCA_001462505.1)	*Aurantiochytrium* FCC1311 [29] (GCA_002897355.1)	*Thraustochytrium* sp. ATCC 26185 [6] (GCA_002154235.1)
Scaffold							
N50	5,831,892	595,797	15,406	367,244	1,342,793	236,568	2139
L50	4	23	1036	68	3	47	2470
N90	1,915,581	144,465	5607	107,216	115,579	51,606	882
L90	12	79	1770	215	42	177	7665
Scaf max length	9,776,036	1,674,554	169,387	1,148,255	19,720,504	1,101,226	43,773
Num of scaffolds	34	322	15,340	215	1847	2232	10,764
Contig							
N50	2,864,487	52,007	13,661	196,455	12,952	22,474	2139
L50	9	230	1141	117	894	527	2469
N90	1,558,441	14,718	1699	57,189	3515	5763	882
L90	21	754	5607	384	3016	1792	7665
Ctg max length	4,854,459	236,430	169,387	729,578	98,696	129,397	10,764
Num of Contigs	67	1608	15,923	848	6833	4504	10,768
Other							
Plasmids	2	–	–	–	–	–	–
Genes	12,392	–	–	–	–	11,520	
Average GC%	44.95%	56.6%	45.19%	57.17%	62.83%	57.13%	64.04%
Total base num	64,068,115	39,089,698	59,569,284	76,822,483	43,429,441	38,943,350	18,099,857

### iTRAQ-based protein identification

**Fig. 2:**
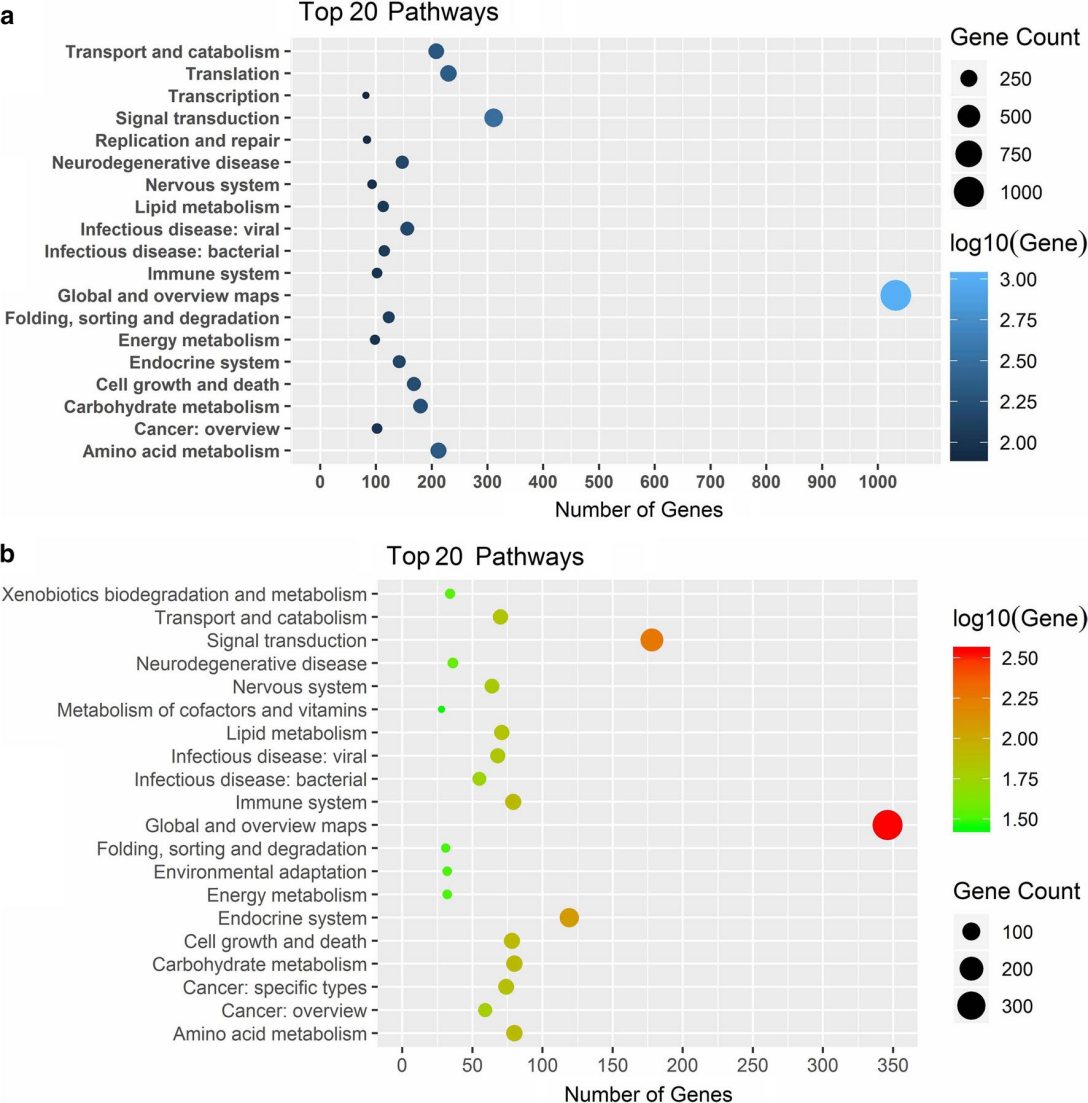
The top 20 KEGG pathways of iTRAQ-identified proteins (**a**) and the top 20 KEGG pathways of differentially expressed genes (**b**)

To identify *Schizochytrium* proteins, six samples of *Schizochytrium* cells were grown at both normal temperatures and low temperatures and subjected to iTRAQ-based proteomic analysis. Tandem mass spectra were searched against the *Schizochytrium* protein database containing the genomics-predicted proteins and transcriptomics-predicted novel proteins. A total of 4008 proteins were quantified (detailed information regarding the iTRAQ-identified proteins is provided in Additional file 2: Table S2), of which 3196 were annotated by the reference genome and 812 were predicted by transcriptomic analysis. Using KEGG pathway analysis, 2939 proteins among the 4008 proteins were annotated in 45 pathways. These identified proteins are involved in global and overview maps, signal transduction, carbohydrate metabolism, lipid metabolism, amino acid metabolism, and energy metabolism, among others. The top 20 KEGG pathways of iTRAQ-identified proteins are shown in Fig. 2a.

### Fatty acid biosynthesis pathway

**Fig. 3:**
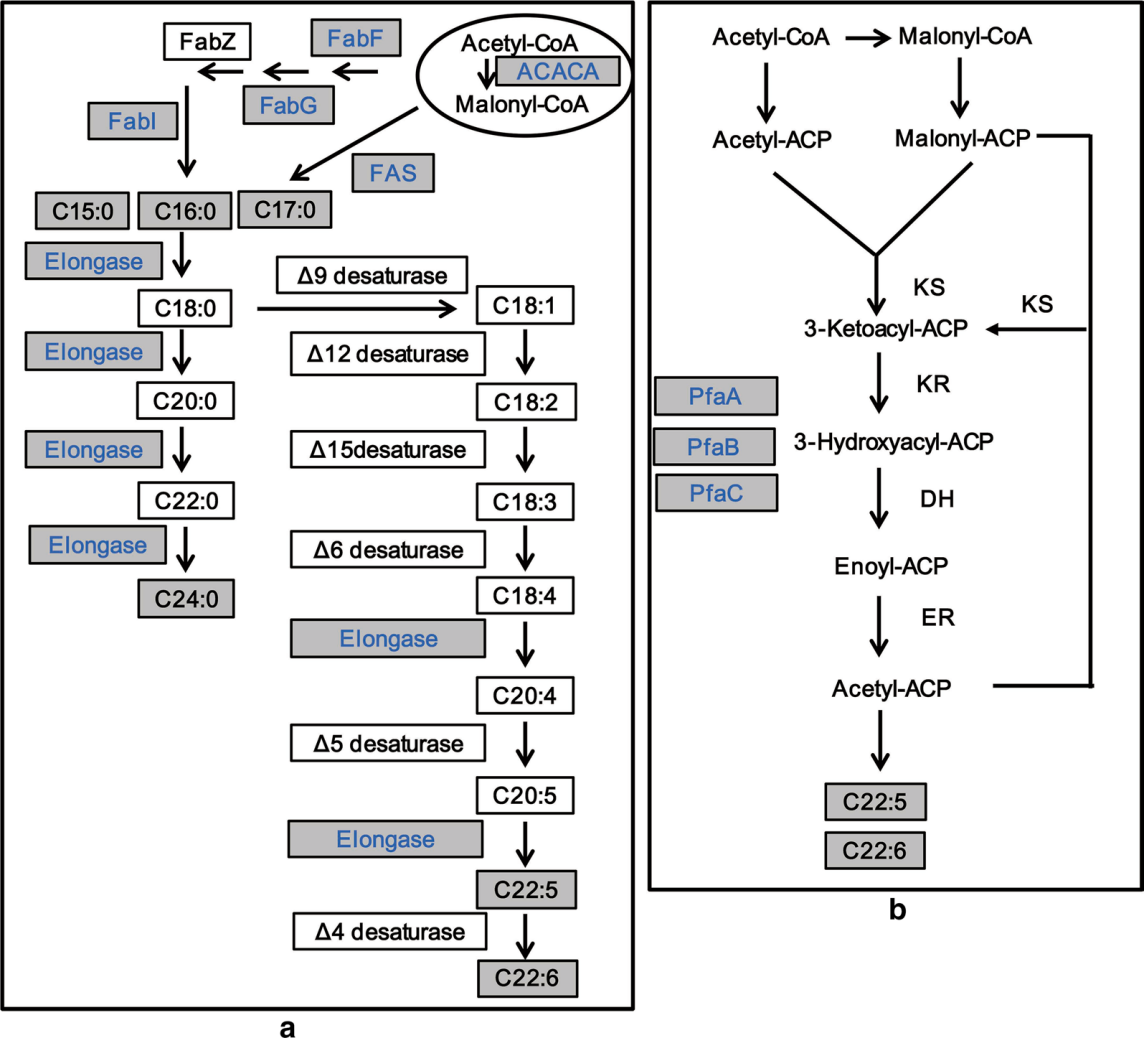
Reconstruction of the fatty acid synthase (FAS, **a**) and polyketide synthase (PKS, **b**) pathways in *Schizochytrium*. Shaded boxes show proteins/fatty acids that were detected from genome annotations/GC analysis; white boxes indicate proteins/fatty acids that were not detected. Proteins that were identified by iTRAQ are indicated in blue. *ACACA* acetyl-CoA carboxylase, *FAS* fatty acid synthase, *FabF* 3-oxoacyl-ACP synthase II, *FabG* 3-oxoacyl-ACP reductase, *FabI* enoyl-ACP reductase, Elongase includes four enzymes: *ELO* long-chain fatty acid elongase, *VLCR* very long-chain 3-oxoacyl-CoA reductase, *HACD* very long-chain (3R)-3-hydroxyacyl-CoA dehydratase, *TECR* trans-2-enoyl-CoA reductase, *PfaA* polyketide synthase subunit A, *PfaB* polyketide synthase subunit B, *PfaC* polyketide synthase subunit C, *KS* beta-ketoacyl-acyl-carrier-protein synthase domain, *KR* ketoacyl reductase domain, *DH* dehydratase domain, *ER* enoylreductase domain

Based on genome annotations and protein identification, the fatty acid biosynthesis pathways of *Schizochytrium*, including the saturated fatty acid synthesis pathway and the polyunsaturated fatty acid synthesis pathway were reconstructed (Fig. 3). Annotations of these proteins and their detailed LC-MS-MS information are provided in Additional file 3: Table S3 and Additional file 2: Table S2, respectively.

As shown in Fig. 3, the FAS pathway and the PKS pathways are, respectively, responsible for saturated fatty acid and polyunsaturated fatty acid synthesis. There are two principal pathways involved in short saturated fatty acid (SSFA) synthesis in *Schizochytrium*. Similar to fungi that use type I fatty acid synthase (FAS) to synthesize short saturated fatty acids in the cytosol, *Schizochytrium* utilizes a single large multifunctional polypeptide to synthesize short saturated fatty acids, which contains 4230 amino acids and shares approximately 99% protein identity with the fatty acid synthase from *Aurantiochytrium mangrovei* (AKV56231.1).

In addition to type I fatty acid synthase, the type II short saturated fatty acid biosynthesis pathway is also believed to be able to synthesize short saturated fatty acids in the cytosol. Type II short saturated fatty acid biosynthesis pathways are usually found in archaea and bacteria, which contain a set of discrete monofunctional enzymes [30]. Most of the genes related to the type II short saturated fatty acid biosynthesis pathway were annotated and identified in *Schizochytrium* (Fig. 3, Additional file 2: Table S2 and Additional file 3: Table S3), except for 3-hydroxyacyl-ACP dehydratase (FabZ). 3-Hydroxyacyl-ACP dehydratase catalyzes the third step of short saturated fatty acid biosynthesis, dehydrating 3-hydroxyacyl-ACP to form trans-2-enoyl-ACP. These results suggest that the type II short saturated fatty acid pathway in *Schizochytrium* sp. TIO01 is incomplete and that type I FAS is responsible for the synthesis of C16:0 and odd-length short saturated fatty acids (C15:0 and C17:0) [31]. In *Schizochytrium*, the long-chain fatty acid elongation process uses C16:0 as a substrate to synthesize long-chain saturated fatty acids (C24:0). This procedure consists of four sequential reactions: condensation, reduction, dehydration, and reduction. Genes participating in the elongation process were all annotated (Additional file 3: Table S3).

Similar to other *Thraustochytriidae* species that use the PKS pathway to synthesize polyunsaturated fatty acids (PUFAs) [32], *Schizochytrium* contains three large multifunctional PKS pathway genes, namely, *pfaA*, *pfaB* and *pfaC*. These three genes are highly homologous (degrees of protein identify > 99%) to proteins from another PUFA-producing species, *Aurantiochytrium* sp. L-BL10. In addition to the PKS pathway, the FAS pathway, which involves serial desaturation and elongation of short saturated fatty acids, is also believed to be able to synthesize PUFAs [6]. Several elongases associated with the FAS pathway for PUFA synthesis were annotated and identified in *Schizochytrium* (Fig. 3, Additional file 2: Table S2 and Additional file 3: Table S3), however, the desaturase associated with the FAS pathway for PUFA synthesis was completely absent. The intermediates of PUFA compounds involved in the FAS pathway for DHA synthesis, such as C18:1, C18:2, C18:3, and C20:3, were also not detected by GC. These results indicate that the FAS pathway for DHA synthesis is incomplete in *Schizochytrium* and that the PKS pathway is responsible for DHA synthesis.

### Transcriptomic profiling under cold temperatures

To improve the understanding of the mechanism by which low temperatures promote DHA accumulation, transcriptomic analysis was performed on six samples at both normal and lower temperatures as described previously. The obtained reads represented an average of 207.11 times the *Schizochytrium* genome length. Among these reads, 11,215 expressed genes were detected, including 9660 genomics predicted genes and 1555 novel predicted genes. A total of 1546 genes among 11,215 expressed genes were significantly associated with the low-temperature response (|fold change|> = 2 and adjusted *p *value <  = 0.001), including 1237 downregulated genes and 309 upregulated genes. Using KEGG pathway analysis, 846 of the 1546 differentially expressed genes were annotated in 45 pathways. These differentially expressed genes were involved in amino acid metabolism, carbohydrate metabolism, lipid metabolism, global and overview maps, signal transduction, the endocrine system, and so on. The top 20 KEGG pathways of differentially expressed genes are shown in Fig. 2b.

### Significant differentially expressed genes involved in fatty acid synthesis

**Fig. 4:**
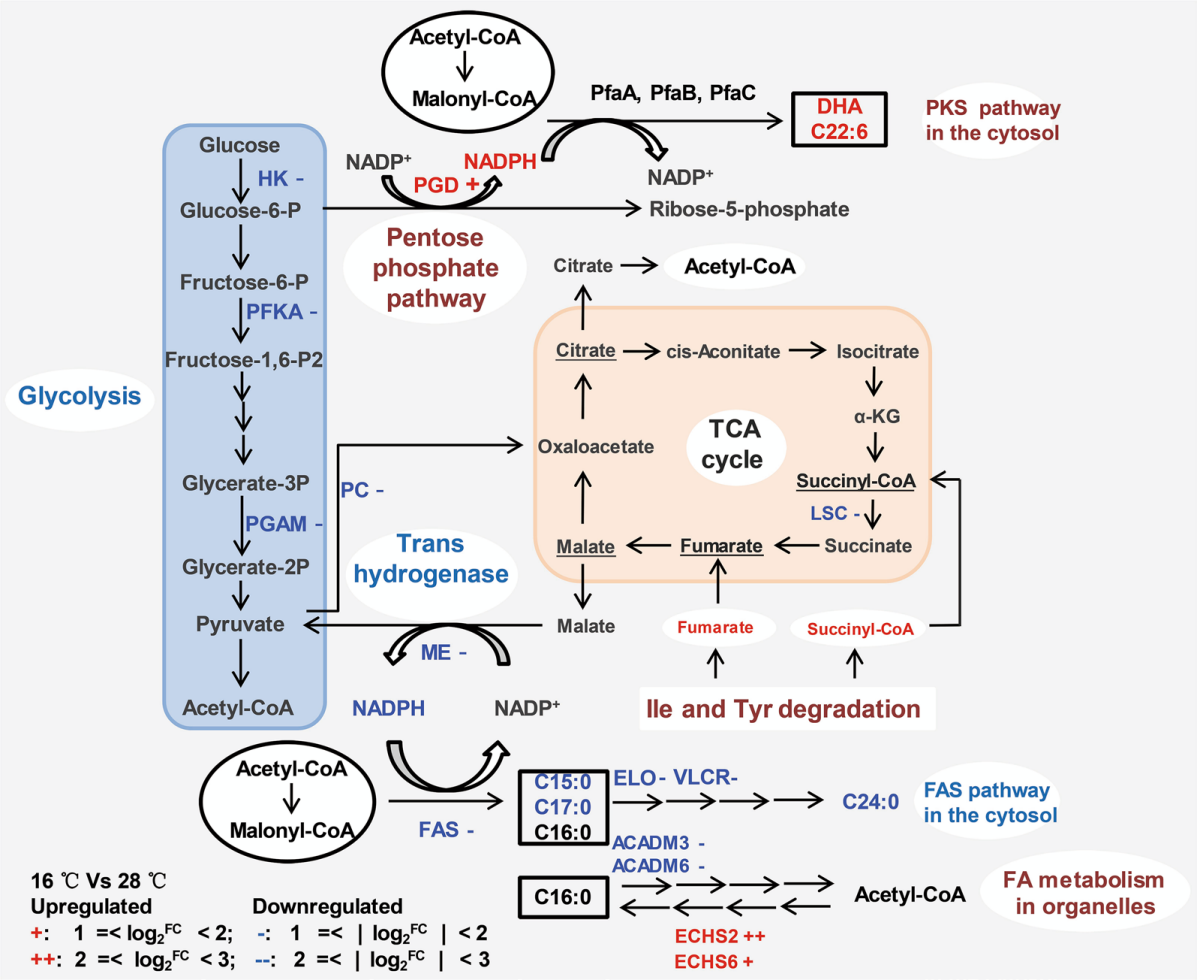
Diagrammatic representation of significantly differentially expressed genes associated with the fatty acid biosynthesis pathways, glycolysis, pentose phosphate pathway and TCA cycle when *Schizochytrium* was cultured under low-temperature conditions. Genes that were differentially regulated (|fold change|> = 2 and adjusted *p* value < 0.001) when *Schizochytrium* was cultured at 16 °C are indicated in red (upregulated) and blue (downregulated). *HK* glycolysis hexokinase, *PFKA* 6-phosphofructokinase, *PGAM* phosphoglycerate mutase, *PC* pyruvate carboxylase, *PGD* glucose-6-phosphate 1-dehydrogenase, *PfaA* polyketide synthase subunit A, *PfaB* polyketide synthase subunit B, *PfaC* polyketide synthase subunit C, *ME* malic enzyme, *FAS* fatty acid synthase, *ELO* long-chain fatty acid elongase, *VLCR* very long-chain 3-oxoacyl-CoA reductase, *ACADM* acyl-CoA dehydrogenase, *ECHS* enoyl-CoA hydratase, *LSC* succinyl-CoA synthetase

As shown in Fig. 1b, *Schizochytrium* exhibited reduced saturated fatty acid content and a preference for increased DHA content when cultured under cold temperatures. Transcriptomic and q-PCR analyses indicated that genes involved in the FAS pathway responsible for saturated fatty acid synthesis (such as those encoding fatty acid synthase (FAS), long-chain fatty acid elongase (ELO), and very-long-chain 3-oxoacyl-CoA reductase (VLCR)) were significantly downregulated (Fig. 4 and Table 2). Gene expression was also significantly downregulated for malic enzyme, which catalyzes the conversion of malate to pyruvate to produce NADPH, which enters exclusively into the FAS pathway for saturated fatty acid synthesis [33, 34]. These results were consistent with the reduced saturated fatty acid content (C15:0, C17:0 and C24:0) at low temperatures. Generally, downregulation of the FAS pathway and malic enzyme is responsible for reduced saturated fatty acid content at low temperatures, however, the production of C16:0 was not affected in this study (Fig. 1b). Further analysis showed that two genes involved in fatty acid metabolism in organelles were significantly regulated (Fig. 4). One of these genes was significantly downregulated and encodes acyl-CoA dehydrogenase (ACADM), which catalyzes the first step of fatty acid beta-oxidation degradation in peroxisomes. The other gene was significantly upregulated and encodes enoyl-CoA hydratase (ECHS), which catalyzes the third step of fatty acid synthesis in mitochondria. These results suggest that fatty acid metabolism in organelles plays an important role in C16:0 synthesis and complements the biosynthesis of C16:0 under low-temperature conditions. Although low temperature has a significant impact on the production of DHA, genes involved in the PKS pathway responsible for the biosynthesis of DHA were not significantly regulated according to the transcriptomic analysis (Fig. 4) and q-PCR results (Table 2). This suggests that the increased DHA content was not a result of the overexpression of PKS pathway genes.

**Table 2:** Transcriptomics and q-PCR analysis of the expression of genes related to fatty acid synthesis

Name	Description	RNA-SEQ 16 °C vs 28 °C (log_2_^Fold Change^)	Q-PCR 16 °C vs 28 °C (log_2_^Fold Change^)
Genes related to SFA synthesis			
ACACA	Acetyl-CoA carboxylase	− 0.99	− 1.14 ± 0.57
FAS	Fatty acid synthase	− 1.52	− 4.02 ± 0.53
ELO	Long-chain fatty acid elongase	− 1.08	− 2.43 ± 0.81
VLCR	Very long-chain 3-oxoacyl-CoA reductase	− 1.07	− 0.91 ± 0.50
ME	Malic enzyme	− 1.03	− 1.11 ± 0.62
Genes related to PUFA synthesis and the pentose phosphate pathway			
PfaA	Polyketide synthase subunit A	− 0.88	0.35 ± 0.22
PfaB	Polyketide synthase subunit B	− 0.79	0.05 ± 0.29
PfaC	Polyketide synthase subunit C	− 0.66	0.12 ± 0.38
PGD	Glucose-6-phosphate 1-dehydrogenase	1.72	3.78 ± 0.57
Genes related to FA metabolism in organelles			
ACADM-3	Acyl-CoA dehydrogenase	− 1.39	− 2.36 ± 0.65
ACADM-5	Acyl-CoA dehydrogenase	− 1.11	− 1.80 ± 0.21
ECHS-2	Enoyl-CoA hydratase	2.47	1.33 ± 0.22
ECHS-6	Enoyl-CoA hydratase	1.54	1.42 ± 0.16
Genes related to glycolysis			
HK	Glycolysis hexokinase	− 1.05	− 0.62 ± 0.35
PFKA	6-Phosphofructokinase	− 1.48	− 0.98 ± 0.21
PGAM	Phosphoglycerate mutase	− 1.22	0.14 ± 0.42
PC	Pyruvate carboxylase	− 1.79	− 1.48 ± 0.031
Genes related to branched-chain amino acid degradation and the TCA cycle			
TAT	Tyrosine aminotransferase	1.77	2.5 ± 0.2
HGD	Homogentisate 1,2-dioxygenase	1.46	1.08 ± 0.19
FAH	Fumarylacetoacetase	1.17	2.23 ± 0.63
OXCT	3-Oxoacid CoA-transferase	1.06	1.12 ± 0.11
ACCAT	Acetyl-CoA C-acetyltransferase	1.58	2.62 ± 0.39
BCATC-1	Ketol-acid reductoisomerase	− 1.12	− 1.36 ± 0.41
BCATC-2	Ketol-acid reductoisomerase	1.28	1.59 ± 0.27
BCAT	Branched-chain amino acid aminotransferase	1.26	2.69 ± 0.14
BCKDH	2-Oxoisovalerate dehydrogenase E1 component	1.11	1.14 ± 0.07
DBT	2-Oxoisovalerate dehydrogenase E2 component	1.52	3.46 ± 0.21
GCDH	Glutaryl-CoA dehydrogenase	1.07	0.52 ± 0.18
LEU	3-Isopropylmalate dehydratase	2.49	3.08 ± 0.15
IVD-2	Isovaleryl-CoA dehydrogenase	2.31	0.91 ± 0.28
IVD-3	Isovaleryl-CoA dehydrogenase	1.66	2.64 ± 0.24
MCCB	3-Methylcrotonyl-CoA carboxylase beta subunit	1.31	2.17 ± 0.27
HMGCS	Hydroxymethylglutaryl-CoA synthase	− 1.19	− 2.18 ± 0.31
LSC	Succinyl-CoA synthetase	− 2.72	− 1.3 ± 0.21

### Significant differentially expressed genes associated with glycolysis and the pentose phosphate pathway

Glycolysis and the pentose phosphate pathway directly produce the substrates (acetyl-CoA and NADPH) for fatty acid synthesis. When *Schizochytrium* was cultured under cold conditions, genes involved in glycolysis such as those encoding glycolysis hexokinase (HK), 6-phosphofructokinase (PFKA), and phosphoglycerate mutase (PGAM) were significantly downregulated (|fold change|> = 2 and adjusted *p* value < 0.001) (Fig. 4 and Table 2), indicating that the glycolysis pathway was inhibited to some extent under cold stress. This result is consistent with the glucose consumption analysis (Fig. 1a).

Glucose-6-phosphate 1-dehydrogenase (PGD), which plays a critical role in microbial and algal lipid accumulation [31, 35], is involved in the pentose phosphate pathway and catalyzes the conversion of gluconate-6P to D-ribulose-5P to produce NADPH. Our findings show that this enzyme was significantly upregulated (Fig. 4 and Table 2). Song et al. and Liu et al. have reported that the NADPH produced by PGD exclusively enters the PKS pathway for PUFA biosynthesis [33, 34]. Taken together, these results indicate that glucose is degraded via the pentose phosphate pathway to produce a relatively large amount of NADPH entering the PKS pathway for DHA biosynthesis at low temperatures.

### Significant differentially expressed genes involved in branched-chain amino acid metabolism and the TCA cycle

**Fig. 5:**
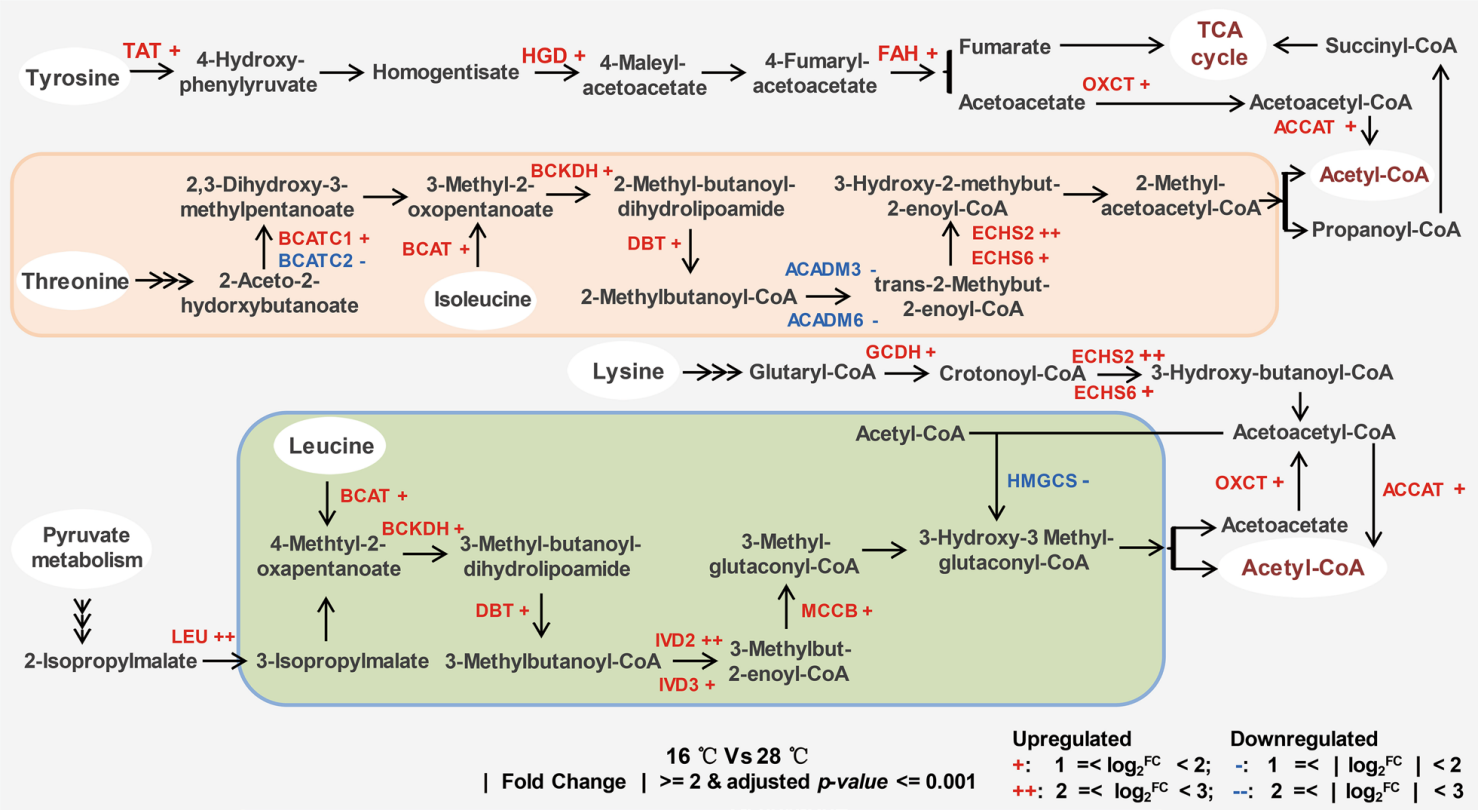
Diagrammatic representation of significantly differentially expressed genes associated with the branched-chain amino acid pathway. Genes that were differentially regulated (fold change >  = 2 and adjusted *p* value <  = 0.001) when *Schizochytrium* was cultured at 16 °C are indicated in red (upregulated) and blue (downregulated). *TAT* tyrosine aminotransferase, *HGD* homogentisate 1,2-dioxygenase, *FAH* fumarylacetoacetase, *OXCT* 3-oxoacid CoA-transferase, *ACCAT* acetyl-CoA C-acetyltransferase, *BCATC* ketol-acid reductoisomerase, *BCAT* branched-chain amino acid aminotransferase, *BCKDH* 2-oxoisovalerate dehydrogenase E1 component, *DBT* 2-oxoisovalerate dehydrogenase E2 component, *ACAMD* acyl-CoA dehydrogenase, *ECHS* enoyl-CoA hydratase, *GCDH* glutaryl-CoA dehydrogenase, *LEU* 3-isopropylmalate dehydratase, *IVD* isovaleryl-CoA dehydrogenase, *MCCB* 3-methylcrotonyl-CoA carboxylase beta subunit, *HMGCS* hydroxymethylglutaryl-CoA synthase

Amino acid degradation leads to the production of acetyl-CoA which is an important substrate for fatty acid synthesis. According to the results of the differentially expressed gene analysis, low temperatures induced significant regulation changes for 20 genes involved in branched-chain amino acid degradation (upregulation of 16 and downregulation of 4) (Fig. 5 and Table 2). Four significantly downregulated genes included genes encoding hydroxymethylglutaryl-CoA synthase (HMGCS), acyl-CoA dehydrogenase (ACADM) and ketol-acid reductoisomerase (BCATC). HMGCS catalyzes the reverse reaction of Leu degradation, leading to the consumption of acetyl-CoA. ACADM participates in Ile degradation and the fatty acid beta-oxidation pathway. There are six copies of ACADM genes in *Schizochytrium*, two of which were significantly downregulated. It is uncertain whether these two downregulated ACADM genes play roles in Ile degradation or in the fatty acid beta-oxidation pathway (Fig. 4). Generally, low temperatures enhanced the degradation of branched-chain amino acids leading to increased production of acetyl-CoA for fatty acid synthesis and TCA intermediate compounds (fumarate and succinyl-CoA).

Most of the genes that participate in the TCA cycle were not significantly regulated at lower temperatures except succinyl-CoA synthetase (LSC). Downregulated succinyl-CoA synthetase reduces the production of the TCA intermediate compound succinate. However, the levels of succinate and the downstream compound fumarate could be increased by enhanced branched-chain amino acid degradation (Tyr and Ile degradation pathways) (Figs. 4 and 5). This indicates that the TCA intermediate compounds (fumarate and succinyl-CoA) produced by branched-chain amino acid degradation could be further degraded to produce the acetyl-CoA precursor citrate through the TCA cycle. Taken together, more acetyl-CoA for fatty acid synthesis could be produced by branched-chain amino acid degradation and the TCA cycle at low temperatures.

## Discussion

*Schizochytrium* species are well known in the field of single-cell oil research for their ability to produce the health-benefitting docosahexaenoic acid. However, the PUFA biosynthesis pathway in *Schizochytrium* has long been controversial [10, 11, 36]. Based on the high-quality genome sequence of *Schizochytrium* sp TIO01, we found that the desaturase involved in the FAS pathway for PUFA synthesis was absent in *Schizochytrium* sp. TIO01, and the FAS pathway and the PKS pathway were, respectively, responsible for SFA and PUFA synthesis. This result was consistent with previous biochemical and genetic studies on *Schizochytrium* [7–9]. The PKS pathway responsible for PUFA synthesis also exists in other *Thraustochytriidae* species including one of the closest relatives of *Schizochytrium*, *Thraustochytrium*. In this species, desaturases involved in the FAS pathway for PUFA synthesis are completely absent. Evidence that the PKS pathway is responsible for PUFA synthesis includes a genomic survey [6], heterologous candidate gene expression [32] and an in vivo feeding experiment [31]. In another close relative*, Aurantiochytrium*, the PKS pathway and the FAS pathway have been proposed to, respectively, produce PUFAs and SFAs based on fatty acid feeding experiments [33]. Taken together, these results suggest that the PKS pathway, not the FAS pathway, is responsible for PUFA synthesis in *Thraustochytriidae*.

Low temperature has a significant impact on PUFA accumulation in many organisms that use the PKS pathway for PUFA synthesis. In this study, we found that low temperatures reduced saturated fatty acid biosynthesis and significantly increased DHA synthesis in *Schizochytrium*. Furthermore, gene expression analysis revealed that FAS pathway genes were significantly downregulated. In contrast, the expression of PKS pathway genes that are responsible for DHA synthesis remained unchanged relative to expression levels at normal temperature. These results suggest that significantly increased DHA content is not attributable to upregulation of PKS pathway genes. This phenomenon also occurs in *Shewanella piezotlerans* and *Photobacterium profundum*, where PUFA content is increased under low-temperature conditions and is not correlated with the overexpression of PKS pathway genes [20, 21]. In one of the closest relatives of *Schizochytrium*, *Aurantiochytrium,* Ma et al. reported that the upregulated PKS pathway genes and downregulated FAS pathway play an important role in promoting DHA accumulation [13, 18]. These results suggest that different mechanisms for regulating PUFA synthesis exist in different organisms.

NADPH and acetyl-CoA are generally accepted as the main substrates for fatty acid biosynthesis. Therefore, fatty acid production is promoted by the overexpression of genes involved in their production (malic enzyme, glucose-6-phosphate dehydrogenase, acetyl-CoA synthetase, citrate lyase, the pyruvate dehydrogenase complex) or by exogenous supplementation with an acetyl-CoA precursor (citrate, acetate) [37]. The two major contributors to NADPH production include malic enzyme and glucose-6-phosphate 1-dehydrogenase (PGD) in the oxidative pentose phosphate pathway. When *Schizochytrium* was cultured at a low temperature, downregulated malic enzyme and upregulated glucose-6-phosphate 1-dehydrogenase (PGD) were detected. Although downregulation of malic enzyme reduces the production of NADPH, recent studies have shown that the NADPH produced by malic enzyme and glucose-6-phosphate 1-dehydrogenase enters the FAS pathway for SFA biosynthesis and the PKS pathway for PUFA biosynthesis, respectively [33, 34]. Downregulation of malic enzyme coupled with decreased saturated fatty acid content, and upregulation of glucose-6-phosphate 1-dehydrogenase coupled with increased polyunsaturated fatty acid content were also observed in this study. Generally, the upregulated pentose phosphate pathway is expected to increase the amount of NADPH entering the PKS pathway for DHA synthesis at low temperatures.

Glycolysis and branched-chain amino acid degradation pathways directly produce acetyl-CoA. When *Schizochytrium* was cultured under low-temperature conditions, glycolysis expression levels were downregulated while branched-chain amino acid degradation pathway levels were upregulated. Although downregulated glycolysis leads to reduced acetyl-CoA production, the upregulated branched-chain amino acid degradation pathway could increase the production of acetyl-CoA. In addition, the downregulated FAS pathway would decrease the consumption of acetyl-CoA, leading to decreased saturated fatty acid content. Generally, a relatively large amount of acetyl-CoA would be able to enter the PKS pathway for DHA synthesis at low-temperature conditions.

Taken together, upregulation of the pentose phosphate pathway and branched-chain amino acid degradation pathway (increasing the production of the substrates for DHA synthesis) and downregulation of the FAS pathway and malic enzyme (reducing the consumption of the substrates) likely play a key role in DHA accumulation in *Schizochytrium* under low temperatures.

## Conclusions

In the present study, multi-omics approaches were used to investigate the effects of low temperatures on DHA biosynthesis and the underlying mechanism in *Schizochytrium* sp. TIO01. Our findings provide evidence that the PKS and FAS pathways are, respectively, responsible for DHA and saturated fatty acid synthesis in *Schizochytrium*. In addition, our work supports the findings that under low-temperature conditions, increased DHA content is not correlated with PKS pathway genes, but rather, increased substrate levels induced by low-temperature conditions entering the PKS pathway.

## Methods

### Growth, glucose consumption, and fatty acid analysis

The *Schizochytrium* sp. TIO01 strain used in this study was isolated previously and deposited in the China General Culture Collection Center (CGMCC no. 4603). Modified YPD medium (glucose 40 g/L, yeast extract 15 g/L, peptone 5 g/L, pH 7.0) with a salinity equivalent to 50% that of seawater was used to culture *Schizochytrium.*

Growth analysis: *Schizochytrium* was cultured overnight at 28 °C in YPD medium and then inoculated into fresh medium at 180 rpm and 28 or 16 °C. Samples were collected at certain intervals, and the optical density was analyzed at 650 nm. The Glucose (GO) Assay Kit (Sigma, GAGO20-1KT) was used to analyze the glucose concentration according to the manufacturer’s protocol. Samples used to analyze the fatty acids were cultured at 28 °C or 16 °C and collected when the OD_650_ reached approximately 0.7. The total lipid content was extracted from freeze-dried samples according to the Bligh–Dyer method [38] and analyzed by an Agilent 7890B GC instrument with H_2_ as the carrier gas using an Agilent J&W CP-Sil 88 column (100 m × 0.25 mm i.d., 0.2 μm film thickness; Agilent Technologies). The operating conditions were as follows: the injector and detector temperatures were kept at 250 °C and 260 °C, respectively; the temperature program was as follows: initial temperature 100 °C for 5 min, increased at 8 °C/min to 180 °C and holding at this temperature for 9 min, followed by increasing at 1 °C/min to 230 °C and holding at this temperature for 15 min. The carrier gas was H_2_ with a flow rate of 40 mL/min and the detector gas flow rate was 40 mL/min for H_2_, and 400 mL/min for air. The FAME mix (C4–C24) consisting of fatty acid standards was purchased from Sigma (cat. no. 18919-1AMP).

### Genome assembly, prediction, and annotation

Genome sequencing sample preparation: *Schizochytrium* was cultured at 28 °C and 180 rpm and collected when the OD_650_ reached approximately 0.7. Total genomic DNA was extracted, and whole-genome shotgun sequencing was performed using the PacBio RS II or Illumina PE250 sequencing platform at the Beijing Genomic Institute (BGI, Shenzhen, China).

Transcriptomics sample preparation for gene prediction: 14 samples were cultured in 7 different media at 28 or 16 °C (the medium components are shown in Additional file 4: Table S4). Harvesting was performed, respectively, when the OD_650_ reached 0.7. Total RNA was extracted and sequencing on each sample was performed on the BGISEQ-500 (PE100) or Illumina platform (PE150) at the BGI, Shenzhen, China.

Genome assembly: Falcon [39] (fc_env_180425) was used to assemble the PacBio long reads multiple times to produce the longest contig N50. A two-step polishing strategy was used to improve accuracy. Initial polishing was performed by Arrow using PacBio long reads, and further corrective polishing was performed by Pilon-v1.22 [40] with highly accurate Illumina PE250 reads. SSPACE-LongRead-v1.1 [41] was used to construct a scaffold using PacBio reads longer than 10,000 bp. Contigs assembled by Platanus-v1.2.4 [42] using Illumina PE250 reads were used to fill the gaps by GMcloser-v1.6.2 [43]. Illumina PE250 reads and transcriptomic reads were analyzed by Bowtie2-v3.4.1 [44] and Histat2-v2.10 [45], respectively, to assess genome completeness. BUSCO-v3.0 [25] (database: eukaryota_odb9) was used to evaluate the completeness of the genome.

Gene prediction and annotation: MAKER-v3.01.02 [46] was used to predict genes by combining results obtained from ab initio prediction (AUGUSTUS-v3.3.1 [47] and SNAP-v2006-07–28 [48]), protein homology search (against protein sequences from the NCBI database: *Aurantiochytrium* sp*.* FCC1311 (GCA_002897355.1), *Blastocystis hominis* (GCA_000151665.1), *Fistulifera solaris* (GCA_002217885.1), *Achlya hypogyna* (GCA_002081595.1), *Phytophthora parasitic* (GCA_000247585.2), and *Thalassiosira pseudonana* (GCA_000149405.2); FGENESH-predicted proteins using the *Phaeodactylum tricornutum* parameters were also used as homologous proteins (https://www.softberry.com/berry.phtml?topic=fgenesh&group=programs&subgroup=gfind)), and transcriptomics (transcripts from 14 diverse conditions were generated using Trinity-v2.6.6 de novo assembly [49] and used as EST evidence). Predicted genes were annotated by alignment to the nonredundant protein sequence (Nr), Kyoto Encyclopedia of Genes and Genomes (KEGG), SwissProt, TrEMBL and InterPro databases using the Basic Local Alignment Search Tool (BLAST) -v2.7.1 + , with an E-value cutoff of 1E-5. Gene Ontology (GO) terms were assigned to the genes using the BLAST2GO pipeline [50].

### iTRAQ labeling, LC–MS/MS and data analysis

iTRAQ labeling and MS/MS: six samples were cultured in YPD medium at two different temperatures (28 and 16 °C). Two samples were collected when the OD_650_ reached 0.7 at 28 °C (28log); two samples were collected when the OD_650_ reached 0.7 at 16 °C (16log); and two samples were collected when the OD_650_ reached 1.4 at 16 °C (16sta). After cell sonication, 100 μg of the proteins was reduced with 10 mM DTT and alkylated with 15 mM iodoacetamide (IAM). Samples were digested at 37 °C with sequencing-grade modified trypsin solution. Desalted peptides were labeled with iTRAQ reagents (6-plex kit, Applied Biosystems, USA) according to the manufacturer's protocol for the 8-plex iTRAQ Kit. Labels 113 and 116 were used to label 16log; 114 and 117 were used to label 16sta; and 115 and 118 were used to label 28log. The iTRAQ-labeled peptide mixture was dissolved and separated into 20 fractions, and each fraction was loaded by an autosampler onto a 2-cm C18 trap column with buffer A (5% ACN, 0.1%, FA, with a linear gradient from 5 to 35%) and buffer B (98% ACN, 0.1% FA) at a flow rate of 300 nL/min, followed by a wash with up to 80% buffer B. Intact peptides were detected in a Thermo Q-Exactive Orbitrap instrument at a resolution of 70,000. The electrospray voltage applied was 1.6 kV. The AGC target was 3e-6 for full MS and 1e-5 for MS2. For MS scans, the m/z scan range was 350 to 2000 Da.

iTRAQ data analysis: MS/MS data were processed using MaxQuant (v. 1.5.3.8). Tandem mass spectra were searched against the *Schizochytrium* protein database, which contains genomics-predicted proteins and transcriptomics-predicted novel proteins. Enzyme specificity was set as full cleavage by trypsin, with two maximum missed cleavage sites permitted. The precursor and fragment ion mass tolerances were 5 ppm and 0.02 Da, respectively. The precursor charged states allowed ranged from 1 to 5. Carbamidomethylation (Cys), iTRAQ 8plex (K) and iTRAQ 8plex (N-term) were set as fixed modifications, whereas oxidation (Met) and acetylation (protein N-terminal) were set as variable modifications. For all experiments, only unique peptides were considered for protein quantification. The estimated false discovery rate (FDR) thresholds for peptides and proteins were specified at a maximum of 1%. The minimum peptide length was set at 6.

### Transcriptomics sample preparation and q-PCR validation

Transcriptomics sample preparation for analyzing gene expression under two different culture conditions: six samples were cultured at 28 and 16 °C in YPD medium. Harvesting was performed when the OD_650_ reached approximately 0.7. Total RNA was extracted and sequencing on each sample was performed on the Illumina platform (PE150) at BGI, Shenzhen, China. Data were analyzed using the Hisat2-StringTie pipeline [45].

q-PCR validation: RNA was extracted using the TRIzol method followed by DNase treatment. The Prime Script II 1st Strand cDNA Synthesis Kit (Takara Biotech Co., Ltd., Dalian, China) was used for cDNA synthesis. q-PCR was performed on a CFX Connect real-time system (Bio-Rad) using Bio-Rad SYBR Green, and 18S rDNA was used as an internal standard; the 2^−ΔΔCT^ method was used to analyze the results [51]. All primers were designed by Primer3Web (https://bioinfo.ut.ee/primer3/), and sequence information is provided in Additional file 5: Table S5.

## Supplementary information

**Additional file 1: Table S1.** Summary of data and read alignments used in genome assembly and assessment.

**Additional file 2: Table S2.** Detailed information regarding the iTRAQ-identified proteins.

**Additional file 3: Table S3.** List of predicted genes involved in fatty acid synthesis.

**Additional file 4: Table S4.** Constitution of media used in RNA_seq sample preparation for protein-coding gene prediction.

**Additional file 5: Table S5.** List of q-PCR primer sequences used in this study.

## Data Availability

The raw sequencing reads used for genome assembly and annotation have been deposited in the SRA (Sequence Read Archive) database under Bioproject ID PRJNA483186. The Whole Genome Shotgun project has been deposited at DDBJ/ENA/GenBank under the accession SMSO00000000. Transcriptomic data for analysis of differential gene expression under the two conditions have been deposited in the SRA database under Bioproject ID PRJNA517294. Protein LC-MS-MS data have been deposited in the IPRoX database (https://www.iprox.org/) under the identifier IPX0001511000.

## Abbreviations

TFA: Total fatty acid
SFA: Saturated fatty acid
SSFA: Short saturated fatty acid
PUFA: Polyunsaturated fatty acid
DHA: Docosahexaenoic acid
DPA: Docosapentaenoic acid
ACACA: Acetyl-CoA carboxylase
FAS: Fatty acid synthase
FabF: 3-Oxoacyl-ACP synthase II
FabG: 3-Oxoacyl-ACP reductase
FabI: Enoyl-ACP reductase
FabZ: 3-Hydroxyacyl-ACP dehydratase
ELO: Long-chain fatty acid elongase
VLCR: Very long-chain 3-oxoacyl-CoA reductase
HACD: Very long-chain (3R)-3-hydroxyacyl-CoA dehydratase
TECR: Trans-2-enoyl-CoA reductase
ME: Malic enzyme
PKS: Polyketide synthase
PfaA: Polyketide synthase subunit A
PfaB: Polyketide synthase subunit B
PfaC: Polyketide synthase subunit C
PGD: Glucose-6-phosphate 1-dehydrogenase
KS: Beta-ketoacyl-acyl-carrier-protein synthase domain
KR: Ketoacyl reductase domain
DH: Dehydratase domain
ER: Enoylreductase domain
HK: Glycolysis hexokinase
PFKA: 6-Phosphofructokinase
PGAM: Phosphoglycerate mutase
PC: Pyruvate carboxylase
ACADM: Acyl-CoA dehydrogenase
ECHS: Enoyl-CoA hydratase
LSC: Succinyl-CoA synthetase
TAT: Tyrosine aminotransferase
HGD: Homogentisate 1,2-dioxygenase
FAH: Fumarylacetoacetase
OXCT: 3-Oxoacid CoA-transferase
ACCAT: Acetyl-CoA C-acetyltransferase
BCATC: Ketol-acid reductoisomerase
BCAT: Branched-chain amino acid aminotransferase
BCKDH: 2-Oxoisovalerate dehydrogenase E1 component
DBT: 2-Oxoisovalerate dehydrogenase E2 component
ACAMD: Acyl-CoA dehydrogenase
ECHS: Enoyl-CoA hydratase
GCDH: Glutaryl-CoA dehydrogenase
LEU: 3-Isopropylmalate dehydratase
IVD: Isovaleryl-CoA dehydrogenase
MCCB: 3-Methylcrotonyl-CoA carboxylase beta subunit
HMGCS: Hydroxymethylglutaryl-CoA synthase
NGS: Next generation sequencing
iTRAQ: Isobaric tags for relative and absolute quantitation
