# Supplementary material for: Low-temperature effects on docosahexaenoic acid biosynthesis in Schizochytrium sp. TIO01 and its proposed underlying mechanism
Source: Biotechnol Biofuels. 2020 Oct 16;13:172. doi: 10.1186/s13068-020-01811-y (PMC7565746; doi:10.1186/s13068-020-01811-y)
Supplement: Supplementary file 1 — Additional file 1: Table S1. Summary of data and read alignments used in genome assembly and assessment. [file 13068_2020_1811_MOESM1_ESM.docx]

**Table S1** Summary of data and read alignments used in genome assembly and assessment

| **Sequence platform** | **Sample** | **Sample Name** | **Num of paired reads** | **Overall alignment rate** | **BaseNum**  **(bp)** | **Total Base (bp)** |
| --- | --- | --- | --- | --- | --- | --- |
| **PacBio RS II**  DNA data | **-** | **-** | - | - | 13,905,179,035 | 13,905,179,035 |
| **Illumina (PE250)**  DNA data | **-** | **-** | 62,457,386 | 99% | 31,228,693,000 | 31,228,693,000 |
| **BGI-500 (PE100)**  RNA data | 1 | 16FA | 20,500,685 | 97.09% | 4,100,137,000 | 57,737,877,800 |
|  | 2 | 28FA | 20,593,949 | 97.46% | 4,118,789,800 |  |
|  | 3 | 16GlyA | 20,483,094 | 97.03% | 4,096,618,800 |  |
|  | 4 | 28GlyA | 20,614,657 | 97.30% | 4,122,931,400 |  |
|  | 5 | 16MSGA | 20,708,328 | 94.87% | 4,141,665,600 |  |
|  | 6 | 28MSGA | 20,762,748 | 97.21% | 4,152,549,600 |  |
|  | 7 | 16NA | 20,464,160 | 96.74% | 4,092,832,000 |  |
|  | 8 | 28NA | 20,544,814 | 97.26% | 4,108,962,800 |  |
|  | 9 | 16PA | 20,612,566 | 97.08% | 4,122,513,200 |  |
|  | 10 | 28PA | 20,401,494 | 97.23% | 4,080,298,800 |  |
|  | 11 | 16NPA | 20,418,743 | 96.65% | 4,083,748,600 |  |
|  | 12 | 28NPA | 20,430,839 | 97.07% | 4,086,167,800 |  |
| **Illumina (PE150)**  RNA data | 13 | 16SA | 14,122,295 | 95.34% | 4,236,688,500 |  |
|  | 14 | 28SA | 13,979,913 | 95.37% | 4,193,973,900 |  |
