# Supplementary material for: Low-temperature effects on docosahexaenoic acid biosynthesis in Schizochytrium sp. TIO01 and its proposed underlying mechanism
Source: Biotechnol Biofuels. 2020 Oct 16;13:172. doi: 10.1186/s13068-020-01811-y (PMC7565746; doi:10.1186/s13068-020-01811-y)
Supplement: Supplementary file 3 — Additional file 3: Table S3. List of predicted genes involved in fatty acid synthesis. [file 13068_2020_1811_MOESM3_ESM.docx]

**Table S3** List of predicted genes involved in fatty acid synthesis

| **Gene ID** | **KO** | **Discription** | **iTRAQ**  **identify** |
| --- | --- | --- | --- |
| SPP02429 | K00667 | FAS, fatty acid synthase, [EC:2.3.1.86] | Y |
| SPP03151 | K01961 | ACACA, acetyl-CoA carboxylase, [EC:6.4.1.2; EC: 6.3.4.14] | Y |
| SPP11327 |  |  | Y |
| SPP09548 | K00645 | MCAT, malonyl CoA-acyl carrier protein transacylase, [EC:2.3.1.39] |  |
| BGI_novel_G001546 | K09458 | FabF, 3-oxoacyl-[acyl-carrier-protein] synthase [EC:2.3.1.179] | Y |
| BGI_novel_G000067 | K00059 | FabG, 3-oxoacyl-[acyl carrier protein] reductase [EC:1.1.1.100] | Y |
| BGI_novel_G000383 |  |  | Y |
| BGI_novel_G001432 |  |  | Y |
| SPP01663 |  |  | Y |
| SPP03170 |  |  | Y |
| SPP06392 |  |  | Y |
| SPP01711 |  |  | Y |
| SPP01854 |  |  | Y |
| SPP04421 |  |  | Y |
| SPP05755 |  |  | Y |
| SPP07858 |  |  | Y |
| SPP08326 |  |  | Y |
| SPP10151 |  |  | Y |
| SPP03221 | K00208 | FabI, enoyl-[acyl-carrier-protein] reductase [EC:1.3.1.9; EC:1.3.1.10] | Y |
| BGI_novel_G000637 | K10246 | ELO, long chain fatty acid elongase, [EC:2.3.1.199] |  |
| SPP05531 |  |  | Y |
| SPP09916 |  |  |  |
| SPP10313 |  |  | Y |
| SPP10648 |  |  |  |
| BGI_novel_G001460 | K10251 | VLCR, very-long-chain 3-oxoacyl-CoA reductase [EC:1.1.1.62; ;EC:1.1.1.330] | Y |
| SPP09611 |  |  | Y |
| SPP09962 |  |  |  |
| SPP10092 |  |  |  |
| SPP06848 | K10703 | HACD , very-long-chain (3R)-3-hydroxyacyl-CoA dehydratase, [EC:4.2.1.134] | Y |
| SPP02062 | K07753 | TECR, trans-2-enoyl-CoA reductase [EC:1.3.1.38] |  |
| SPP03466 |  | PfaA, polyketide synthase subunit A | Y |
| SPP03420 |  | PfaB, polyketide synthase subunit B | Y |
| BGI_novel_G000206 |  | PfaC, polyketide synthase subunit C | Y |
