# Supplementary material for: Low-temperature effects on docosahexaenoic acid biosynthesis in Schizochytrium sp. TIO01 and its proposed underlying mechanism
Source: Biotechnol Biofuels. 2020 Oct 16;13:172. doi: 10.1186/s13068-020-01811-y (PMC7565746; doi:10.1186/s13068-020-01811-y)
Supplement: Supplementary file 4 — Additional file 4: Table S4. Constitution of media used in RNA_seq sample preparation for protein-coding gene prediction. [file 13068_2020_1811_MOESM4_ESM.docx]

**Table S4** Constitution of media used in RNA_seq sample preparation for protein-coding gene prediction

| **Medium** | **SA** (g/L) | **FA**  (g/L) | **GlyA**  (g/L) | **MSGA**  (g/L) | **NA**  (g/L) | **PA**  (g/L) | **NPA**  (g/L) | **Trace**  **elements**  (mg/L) | |
| --- | --- | --- | --- | --- | --- | --- | --- | --- | --- |
| **Glycerol** | - | - | 100 | - | - | - | - | **CaCl_2_** | 50 |
| **Glucose** | 40 | 40 | - | 40 | 40 | 40 | 40 | **MnCl_2_** | 5.2 |
| **Yeast Extract** | 15 | 15 | 14 | - | 0.1 | - | - | **ZnSO_4_** | 5.2 |
| **Peptone** | 5 | 5 | - | - | - | - | 0 | **CuSO_4_** | 0.8 |
| **(NH_4_)_2_SO4** | - | - | 1 | - | - | 1 | 0.1 | **Na_2_MoO_4_** | 0.016 |
| **Monosodium  Glutamate** | - | - | - | 15 | - | 14 | - | **NiSO_4_** | 0.8 |
| **KH_2_PO_4_** | - | - | 3 | 3 | 3 | 0.1 | 0.1 | **FeSO_4_** | 0.01 |
| **Na_2_SO_4_** | - | - | 12 | 12 | 12 | 12 | 12 | **CoCl_2_** | 0.066 |
| **MgSO_4_** | - | - | 5 | 5 | 5 | 5 | 5 | **Thiamine** | 0.76 |
| **K_2_SO_4_** | - | - | 7 | 7 | 7 | 7 | 7 | **vitamin B_12_** | 1.2 |
| **KCl** | - | - | 2 | 2 | 2 | 2 | 2 | **Ca_1/2_**  **Pantothenate** | 25.6 |
| **Trace elements** | - | - | + | + | + | + | + |  |  |

* SA: 50% sea water, FA: fresh water, GlyA: glycerol as a carbon source, MSGA: monosodium glutamate as a nitrogen source, NA: lack of nitrogen source, PA: lack of phosphorus source, NPA: lack both of nitrogen and phosphorus.
